# Manifestations of Aging in Virtual Reality Implementation of Rod and Frame Test

**DOI:** 10.1093/geroni/igab046.2610

**Published:** 2021-12-17

**Authors:** Michal Adamski, Miroslaw Latka, Anna Latka, Bruce West

**Affiliations:** 1 Wrocław University of Science and Technology, Wrocław, Dolnoslaskie, Poland; 2 Wroclaw University of Science and Technology, Wroclaw, Dolnoslaskie, Poland; 3 Opole Neuropsychiatric Hospital, Opole, Opolskie, Poland; 4 Army Research Office, Research Triangle Park, North Carolina, United States

## Abstract

Senior adults’ reliance on the visual frame of reference for spatial orientation is a manifestation of an age-related shift in cognitive style from field independence to field dependence. We implemented a virtual reality rod and frame test (VR-RFT) to assess visual field dependence (VFD) in n=39 young adults (20-30 years old) and n=43 seniors (60 years old and above). The subjects were asked to determine subjective visual vertical (SVV) for 19 angles of frame tilt (running from -45 degrees to 45 degrees in steps of 5 degrees). The strong VFD of seniors was manifested not only by the increased error in the determination of SVV (SVVE) but also in its distribution. For small and large frame tilt angles, seniors’ SVVE skewness and kurtosis were greater than those of young adults. The SVVE median dependence on frame tilt may be accounted for with a phenomenological model whose two parameters describe the strengths of primary (P) and secondary (S) visual attractors which subjects use to infer SVV: the edges of the frame and its imaginary diagonals. For young adults, these parameters were: PY=14.91 and SY=12.51. For seniors, we observed an over 50% increase in the strength of the primary attractor PS=26.31 while the strength of the secondary one was only weakly affected by aging: SS=13.74. We demonstrate that the asymmetry between the strength of attractors significantly contributes to SVVE made by seniors at large frame tilts. We hypothesize that a variant VR-RFT may be used in rehabilitation to reduce excessive VFD.

